# Associations of adverse and protective childhood experiences with thwarted belongingness, perceived burdensomeness, and suicide risk among sexual minority men

**DOI:** 10.1017/S0033291722002823

**Published:** 2023-09

**Authors:** Raymond L. Moody, Joseph A. Carter, Ali Talan, K. Marie Sizemore, Stephen T. Russell, H. Jonathon Rendina

**Affiliations:** 1 Department of Epidemiology, Columbia University Mailman School of Public Health, 772 West 181st Street, New York, NY 10032, USA; 2 Department of Psychology, City University of New York Graduate Center, Health Psychology and Clinical Sciences Program, New York, NY, USA; 3 Whitman-Walker Institute, Washington, DC, USA; 4 Department of Psychiatry, Rutgers University Institute for Health, Health Care Policy, and Aging, New Brunswick, NJ, USA; 5 Department of Human Development and Family Sciences, University of Texas at Austin School of Human Ecology, Austin, TX, USA; 6 Department of Epidemiology, Milken Institute School of Public Health, George Washington University, and Whitman-Walker Institute, Washington, DC, USA

**Keywords:** ACES, PACES, thwarted belongingness, perceived burdensomeness, suicide, sexual minority men

## Abstract

**Background:**

Sexual minority men (SMM) experience higher suicidal ideation and suicide attempts than the general population. We examined the associations of adverse childhood experiences (ACES) and protective and compensatory childhood experiences (PACES) with suicidal ideation and suicide attempts in adulthood via thwarted belongingness and perceived burdensomeness among SMM.

**Methods:**

Data are from the *UNITE* study, a national longitudinal cohort study of HIV-negative SMM from the 50 U.S. states and Puerto Rico. Between 2017 and 2019, participants (*N* = 6303) completed web-based assessments at baseline and 12-month follow-up. ACES and PACES occurring before the age of 18, and current symptoms of thwarted belongingness and perceived burdensomeness were assessed at baseline. Past-week suicidal ideation and past-year suicide attempt were assessed at follow-up.

**Results:**

424 (6.7%) participants reported past-week suicidal ideation and 123 (2.0%) reported a past-year suicide attempt. The results of our multivariate model suggest that each additional adverse childhood experience was prospectively associated with 14% higher odds of past-week suicidal ideation (AOR = 1.14, 95% CI 1.09–1.19) and 19% higher odds of past-year suicide attempt (AOR = 1.19, 95% CI 1.11–1.29). Each additional protective childhood experience was prospectively associated with 15% lower odds of past-week suicidal ideation (AOR = 0.85, 95% CI 0.81–0.90) and 11% lower odds of past-year suicide attempt (AOR = 0.89, 95% CI 0.82–0.98). Perceived burdensomeness partially mediated these prospective associations.

**Conclusion:**

To reduce suicide, screening and treating perceived burdensomeness among SMM with high ACES may be warranted. PACES may decrease perceived burdensomeness and associated suicide risk.

## Introduction

Suicide is the 10^th^ leading cause of death in the United States (Hedegaard, Curtin, & Warner, 2021), and sexual minority men (SMM) remain disproportionately at risk for suicidal ideation and suicide attempts compared to heterosexual men (Hottes, Bogaert, Rhodes, Brennan, & Gesink, 2016; King et al., 2008; Ploderl et al., 2013; Salway et al., 2021; Salway, Ploderl, Liu, & Gustafson, 2019). Specifically, lifetime rates of suicidal ideation are estimated to be three times higher among SMM than in the general population (Luo, Feng, Fu, & Yang, 2017). Data from the 2015–2017 US National Survey on Drug Use and Health revealed that rates of past-year suicide attempts were highest for heterosexual men between the ages of 18 and 20 and steadily declined as these men aged, whereas rates were significantly higher for SMM across all age groups; even the lowest rate of past-year suicide attempts among SMM (ages 50+) exceeded the highest rates observed among heterosexual men (ages 18–20; Salway et al., 2021).

The Interpersonal-Psychological Theory of Suicide posits that thwarted belongingness and perceived burdensomeness are the most proximal mental states that lead to suicidal ideation and suicide attempts across the lifespan (Chu et al., 2017; Joiner et al., 2009). Accordingly, humans possess a fundamental need for belonging. When this need is unmet (i.e. thwarted belongingness), individuals experience a range of adverse health outcomes, including a heightened risk for suicide (Fassberg et al., 2012; Holt-Lunstad, Smith, Baker, Harris, & Stephenson, 2015). Thwarted belongingness is defined by aspects of social disconnectedness, including loneliness and the absence of reciprocal care (Van Orden, Cukrowicz, Witte, & Joiner, 2012; Van Orden et al., 2010). Perceived burdensomeness is defined by aspects of interpersonal conflict, including self-hatred and self-perceptions of being a liability toward others (Van Orden et al., 2010, 2012). Specifically, individuals with high perceived burdensomeness perceive that their death has more value than their life to others. Importantly, thwarted belongingness and perceived burdensomeness are dynamic constructs modifiable through clinical intervention (Van Orden et al., 2010, 2012), suggesting that research identifying the influence of these psychosocial mechanisms on suicide risk is necessary to inform suicide prevention interventions.

Although prior research has demonstrated that perceived burdensomeness and, to a lesser extent, thwarted belongingness are associated with risk for suicide, SMM are underrepresented in this research, limiting the generalizability (Chu et al., 2017). For SMM, thwarted belongingness and perceived burdensomeness are likely influenced by sexual minority status (Hatzenbuehler, 2009). For example, sexual minority individuals are more likely to experience family rejection and other forms of sexual orientation-related discrimination that disrupt access to social support (Hatzenbuehler, 2011). Consequently, SMM may find it challenging to connect with an accepting community where they feel like they belong and can be their authentic self, impacting thwarted belongingness. Additionally, sexual minority stigma is a unique stressor that places an additional burden on sexual minority individuals (Meyer, 2013). However, the burden of sexual minority stigma may extend beyond the sexual minority individual to impact the lives of those associated with the sexual minority individual (Hilton & Szymanski, 2011). For example, a sexual minority individual may believe that they are a burden to their parent because their parent is embarrassed by their sexual orientation and makes efforts to avoid discussing the subject with community members. As such, there remains a need for studies that specifically examine the influence of thwarted belongingness and perceived burdensomeness on suicide risk among SMM and other sexual minority populations.

Few studies have examined the associations between thwarted belongingness, perceived burdensomeness, and suicide risk in sexual minority populations. Prior research suggests that perceived burdensomeness, but not thwarted belongingness, is higher among sexual minority college students than among heterosexual college students (Hill & Pettit, 2012; Pate & Anestis, 2020). One potential explanation may be that transitioning to college disrupts social networks for most students but for sexual minority students, it may also be the beginning of the coming out process. While all students attempt to establish new social supports, sexual minority students may be navigating the new burdens their sexual orientation may place on their existing relationships (Puckett, Woodward, Mereish, & Pantalone, 2015). Cross-sectional research suggests that perceived burdensomeness is associated with suicidal ideation and suicide attempts in samples of sexual minority youth and adults (Baams, Grossman, & Russell, 2015; Hill & Pettit, 2012; Ploderl et al., 2013, 2014; Woodward, Wingate, Gray, & Pantalone, 2014). This research has failed to demonstrate consistent associations between thwarted belongingness and suicidality despite research suggesting this is a significant predictor of suicide in the general population (Chu et al., 2017). Research has not yet examined the effects of thwarted belongingness and perceived burdensomeness in prospective studies of suicide risk among SMM. This research is needed to understand how thwarted belongingness and perceived burdensomeness may influence future risk for suicide. Further, research is required in order to identify factors that contribute to thwarted belongingness and perceived burdensomeness and potentially increase the future risk for suicide via these psychosocial mechanisms.

Adverse childhood experiences (ACES) are complex and potentially traumatic early stressors occurring before age 18 that can disrupt developmental processes and affect health trajectories and outcomes throughout adulthood (Petruccelli, Davis, & Berman, 2019). ACES include neglect, abuse, and other household dysfunction (e.g. parental incarceration; Andersen & Blosnich, 2013). Sexual minority individuals experience higher rates of some ACES, including childhood physical and sexual assault and emotional maltreatment, and a higher cumulative burden of ACES overall compared to their heterosexual peers (Andersen & Blosnich, 2013; Austin, Herrick, & Proescholdbell, 2016). Studies have demonstrated that ACES are associated with a higher risk of adverse physical and mental health outcomes in adulthood, including depression, substance use, and suicidality (Austin et al., 2016; Sahle et al., 2021; Thompson, Kingree, & Lamis, 2019). As such, ACES likely contribute to the high morbidity of these health outcomes in SMM (Andersen, Zou, & Blosnich, 2015; Austin et al., 2016; Choi, DiNitto, Marti, & Segal, 2017).

Compared to ACES, less attention has been given to the influence of protective and compensatory childhood experiences (PACES) on suicide risk in adulthood. PACES are advantageous experiences that occur before age 18 and offer protection and resilience against negative physical and mental health outcomes (Crandall et al., 2019). PACES include supportive relationships, community, educational, and household resources, and structure and fair rules at the home (Morris et al., 2018). Population studies of sexual and gender minority youth indicate that connectedness to supportive school environments, communicative and affirming family environments, and policies that prohibit sexual orientation and gender-based harassment and discrimination are correlated with lower rates of suicidal ideation and suicide attempts (Hatzenbuehler & Pachankis, 2016; Raifman, Moscoe, Austin, & McConnell, 2017; Ross-Reed, Reno, Penaloza, Green, & FitzGerald, 2019). Studies of adolescent populations more broadly suggest that some of these factors may offer protection against suicide and other adverse health outcomes regardless of the level of exposure to ACES (Lensch et al., 2021). Still, it is unknown what impact the accumulation of PACES may have on the risk for suicide in adulthood among SMM.

ACES and PACES may impact suicide and other health outcomes in adulthood by influencing psychosocial developmental processes associated with adult health (Crandall et al., 2019). For example, a longitudinal study that followed a cohort of adolescents over ten years into young adulthood found that ACES were associated with poorer self-regulation and PACES were associated with better self-regulation and lower shame (Rollins & Crandall, 2021). Further, the effects of ACES and PACES on anxiety, depression, and substance use were mediated by self-regulation. Research suggests that childhood maltreatment is associated with anxious and avoidant interpersonal behavior and maladaptive schemas (e.g. failure, social isolation) in adulthood (Ihme et al., 2022; Pilkington, Bishop, & Younan, 2021). This research suggests that ACES would be associated with higher thwarted belongingness and perceived burdensomeness. In contrast, PACES are expected to be associated with lower thwarted belongingness and perceived burdensomeness as previous research has demonstrated these experiences are associated with optimism, improve interpersonal behavior, and help foster the development of healthy relationships (Narayan, Rivera, Bernstein, Harris, & Lieberman, 2018; Poole, Dobson, & Pusch, 2017).

Research has demonstrated that ACES are an important risk factor for suicide and other adverse health outcomes in adulthood. However, few studies have also included PACES when examining the impact of ACES on adult health outcomes. The present study sought to investigate thwarted belongingness and perceived burdensomeness as mechanisms mediating the associations of ACES and PACES with suicidality in a longitudinal study of SMM. This research is needed to understand how ACES and PACES, experiences that occur during childhood and adolescence, impact suicide risk in adulthood. Findings from this research may improve psychosocial interventions to reduce suicide risk among vulnerable SMM. Prior analysis testing the associations of thwarted belongingness and perceived burdensomeness on suicidality in sexual minority samples have been cross-sectional. It is unknown whether these are significant risk factors for suicide among SMM. Based on this limited research, we hypothesized that ACES would be positively associated with thwarted belongingness and perceived burdensomeness, and PACES would be negatively associated with thwarted belongingness and perceived burdensomeness. Further, we hypothesized that ACES would be positively associated with suicidal ideation and suicide attempts via higher perceived burdensomeness, and PACES would be negatively associated with suicidal ideation and suicide attempts via lower perceived burdensomeness. Finally, we hypothesized that thwarted belongingness would not be associated with suicidal ideation or suicide attempts.

## Methods

Data are from the baseline and 12-month follow-up assessments of *UNITE*, a national longitudinal cohort study of SMM aimed at understanding factors associated with HIV infection (Rendina et al., 2021). Data were collected between 2017 and 2019.

### Participants and procedures

Participants were recruited via advertisements on geosocial networking apps, social media sites, and email. Participants were eligible if they were (1) age 16 or older; (2) male-identified, including transgender men; (3) identified with a sexual minority status (i.e. gay, bisexual, queer); (4) HIV-negative or of unknown status; (5) reported using a mobile app to find a sexual partner(s) in the past six months; (6) engaged in sexual HIV risk behavior in the past six months; (7) lived in the U.S. states or Puerto Rico; and (8) had a mailing address where they could receive packages. Participants were excluded if they did not complete and return the HIV antibody test, had an HIV-positive test result at baseline, or were identified as duplicates based on the contact information provided. All participants provided informed consent before enrollment. Enrolled participants received a link via email to complete online surveys, available in both English and Spanish, at baseline and follow-up. Study procedures also included annual at-home HIV testing. Participants were compensated with a $25 Amazon electronic gift card for completing the annual survey and HIV testing. The City University of New York Institutional Review Board approved all study procedures.

### Measures

#### Aces

The 10-item ACES questionnaire (Felitti et al., 1998) was administered at baseline and retrospectively assessed a range of childhood adversities, before the age of 18, including verbal abuse and physical abuse by a parent or other adult in the household (e.g. insulted, humiliated, slapped, hit), sexual abuse by an adult or person at least five years older, physical and emotional neglect (e.g. no one made you feel special or important, not enough to eat), and household dysfunction (e.g. parental separation, incarcerated household member). Response options were ‘yes’ or ‘no,’ and a total score was calculated based on the sum of yes responses to all 10 items.

#### PACES

The 10-item PACES questionnaire (Morris et al., 2018) was administered at baseline and retrospectively assessed a range of positive and supportive childhood experiences, before the age of 18, including social support (e.g. best friend, mentor), educational and community resources (e.g. organized sports, civic groups, volunteer organizations), and structure and safety in the household (e.g. fair rules, routine, clean household). Response options were ‘yes’ or ‘no,’ and a total score was calculated based on the sum of yes responses to all 10 items.

#### Thwarted belongingness and perceived burdensomeness

Participants completed the 15-item Interpersonal Needs Questionnaire at baseline (Van Orden et al., 2012). This questionnaire measured constructs of thwarted belongingness, including loneliness and the absence of reciprocal care (e.g. ‘I rarely interact with people who care about me,’ 'I am close to other people'). The questionnaire also measured constructs of perceived burdensomeness, including self-hatred and liability towards others (e.g. 'I think I am a burden to society,' 'the people in my life would be better off if I were gone'). Response options were rated on a 7-point Likert-type ranging from 1 (*Not at all true for me*) to 7 (*Very true for me*). The thwarted belongingness and perceived burdensomeness subscales were validated in prior research of young and older adults (Van Orden et al., 2012). In these validation studies, analyses supported convergent and divergent validity for both subscales. For example, thwarted belongingness was positively correlated with the UCLA Loneliness scale, and perceived burdensomeness was negatively associated with the self-liking subscale or the Self-Competence and Self-Liking Scale. In the current study, the thwarted belongingness subscale score was the sum of responses across nine items and showed strong internal consistency (*α* = 0.91). The perceived burdensomeness subscale score was the sum of responses across six items and showed strong internal consistency (*α* = 0.94).

#### Depression

The 10-item Center for Epidemiological Studies – Depression self-report scale (Radloff, 2016) was administered at baseline and measured symptoms of depression within the past three months. Response options were rated on a 4-point Likert-type scale and ranged from 0 (*Rarely or none of the time*) to 3 (*Most or all of the time*), and a total scale score was used as an indicator of depression (*α* = 0.87).

#### Suicidality

Participants completed an 8-item self-harm questionnaire at follow-up based on the Ask Suicide-Screening Questionnaire Toolkit (Horowitz et al., 2020). The study examined single items measuring suicidal ideation in the past week (i.e. ‘In the past week, have you been having thoughts of killing yourself?’); as well as reported suicide attempts (i.e. ‘In the past 12 months, have you tried to kill yourself?’). Response options for these items were dichotomous, with ‘yes’ or ‘no’ responses.

### Analysis plan

We compared rates of suicidal ideation and attempts at follow-up by baseline demographics using chi-square tests of independence (SPSS version 28.0). Next, we estimated bivariate associations using Pearson correlations and independent sample *t* tests. Finally, we used default maximum likelihood estimation to examine our multivariate logistic path model in Mplus (version 8.0). The benefit of this approach is that we were able to estimate multiple regressions simultaneously and our hypothesized indirect pathways. In the model, baseline thwarted belongingness and perceived burdensomeness were regressed on retrospective accounts of ACES and PACES. Suicidal ideation and suicide attempts at follow-up were regressed on baseline thwarted belongingness, perceived burdensomeness, and retrospective accounts of ACES and PACES. Our model was adjusted for depression by including it as a mediator. All paths were adjusted for baseline age, sexual identity, employment status, income, and education. We used model constraints to estimate the indirect associations and combined associations (i.e. direct and indirect pathways) of ACES and PACES with suicidal ideation and suicide attempt via thwarted belongingness, perceived burdensomeness, and depression. We report standardized regression coefficients and adjusted odds ratios (AOR) where appropriate. We estimated 95% confidence intervals (CI) and statistical significance using bootstrapping with 10 000 replications. We used data from participants who completed the baseline and follow-up (*N* = 6303). We ran a series of more conservative models for sensitivity analyses based on missing data, and the pattern of coefficients and significance did not meaningfully alter the conclusions.

## Results

### Sample characteristics and bivariate analyses

Baseline demographics of the analytical sample (*N* = 6303) and bivariate associations with past-week suicidal ideation and a past-year suicide attempt at follow-up are shown in Table 1. The mean age of the sample was 33.23 (s.d. = 11.45), and most participants identified as gay (81.9%). The sample was diverse regarding race and ethnicity, and socioeconomic indicators. At follow-up, 424 participants (6.7%) reported past-week suicidal ideation, and 123 (2.0%) reported at least one suicide attempt in the past year. Participants who identified as queer at baseline were more likely to report suicidal ideation at follow-up compared to gay and bisexual identified participants (14.3% *v.* 6.4% and 7.4%, respectively). Participants with a college degree at baseline were less likely to report suicidal ideation (5.5%) or suicide attempt (1.0%) at follow-up compared to those with less education. Similarly, participants with full-time employment at baseline were less likely to report suicidal ideation (5.7%) and suicide attempt (1.5%) at follow-up compared to those with less than full-time employment. Those earning an income of $ 50 000 or more at baseline were less likely to report suicidal ideation (3.6%) and suicide attempt (0.5%) at follow-up compared to those earning less than $ 50 000 a year. Finally, participants aged 18–24 at baseline reported the highest risk for suicidal ideation (9.0%) and a suicide attempt (2.8%) at follow-up compared to the other age groups.

**Table 1.** Associations between baseline demographic characteristics of the sample and suicidal ideation and suicide attempt at 12-month follow-up

		Suicidal ideation (past week)^a^			Suicide attempt (past year)^a^		
	Full sample	No	Yes		No	Yes	
	*n* (%)	*n* (%)	*n* (%)	*X^2^* (*p*)	*n* (%)	*n* (%)	*X^2^* (*p*)
*Demographic characteristics*		5879 (93.3)	424 (6.7)		6180 (98)	123 (2.0)	
Sexual orientation							
Gay	5165 (81.9)	4837 (82.3)	328 (77.4)	17.74 (<0.001)	5074 (82.1)	91 (74.0)	5.38 (0.068)
Bisexual	963 (15.3)	892 (15.2)	71 (16.7)		936 (15.1)	27 (22.0)	
Queer	175 (2.8)	150 (2.6)	25 (5.9)		170 (2.8)	5 (4.1)	
Race/Ethnicity							
Black/African American	657 (10.4)	615 (10.5)	42 (9.9)	3.66 (0.454)	639 (10.3)	18 (14.6)	3.12 (0.539)
Hispanic/Latino	1459 (23.1)	1361 (23.2)	98 (23.1)		1433 (23.2)	26 (21.1)	
White	3376 (53.6)	3139 (53.4)	237 (55.9)		3311 (53.6)	65 (52.8)	
Multiracial	380 (6.0)	353 (6.0)	27 (6.4)		372 (6.0)	8 (6.5)	
Other	431 (6.8)	411 (7.0)	20 (4.7)		425 (6.9)	6 (4.9)	
Education							
HS Diploma, GED, or less	851 (13.5)	781 (13.3)	70 (16.5)	13.46 (0.001)	825 (13.3)	26 (21.1)	24.40 (<0.001)
Some college or enrolled	2601 (41.3)	2403 (40.9)	198 (46.7)		2533 (41.0)	68 (55.3)	
College degree or more	2851 (45.2)	2695 (45.8)	156 (36.8)		2822 (45.7)	29 (23.6)	
Employment							
Unemployed	1034 (16.4)	939 (16.0)	95 (22.4)	19.23 (<0.001)	995 (16.1)	39 (31.7)	21.93 (<0.001)
Part-time	1251 (19.8)	1152 (19.6)	99 (23.3)		1228 (19.9)	23 (18.7)	
Full-time	4018 (63.7)	3788 (64.4)	230 (54.2)		3957 (64)	61 (49.6)	
Income							
Less than $ 20 000	2011 (31.9)	1817 (30.9)	194 (45.8)	54.52 (<0.001)	1954 (31.6)	57 (46.3)	28.46 (<0.001)
$ 20 000 to $ 49 999	2605 (41.3)	2435 (41.4)	170 (40.1)		2547 (41.2)	58 (47.2)	
$ 50 000 or more	1687 (26.8)	1627 (27.7)	60 (14.2)		1679 (27.2)	8 (6.5)	
Relationship status							
Single	4545 (72.1)	4229 (71.9)	316 (74.5)	1.32 (0.250)	4460 (72.2)	85 (69.1)	0.56 (0.453)
Partnered	1758 (27.9)	1650 (28.1)	108 (25.5)		1720 (27.8)	38 (30.9)	
Age							
18 to 24	1488 (23.6)	1354 (23.0)	134 (31.6)	23.14 (<0.001)	1446 (23.4)	42 (34.1)	11.89 (0.008)
25 to 34	2555 (40.5)	2379 (40.5)	176 (41.5)		2503 (40.5)	52 (42.3)	
35 to 49	1526 (24.2)	1445 (24.6)	81 (19.1)		1504 (24.3)	22 (17.9)	
50 or more	734 (11.6)	701 (11.9)	33 (7.8)		727 (11.8)	7 (5.7)	

*N* = 6303.

a Suicidal ideation (past week) and suicide attempt (past year) are dichotomous variables (Yes/No).

Descriptive statistics (mean, median, standard deviation, and interquartile range) and bivariate associations for our predictor and outcome variables are provided in online Supplementary Table (see online Supplementary Table S1).

### Multivariate path analysis

**Fig. 1.**
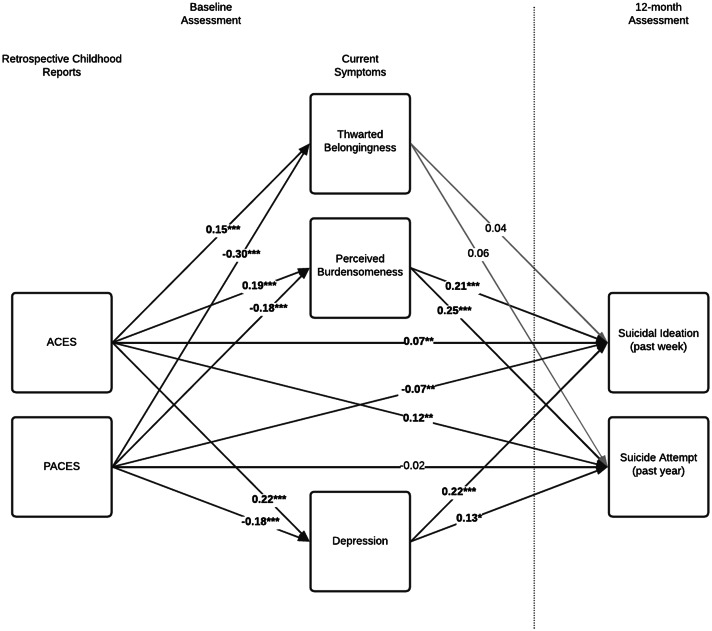
Model depicting the direct associations among ACES, PACES, thwarted belongingness, perceived burdensomeness, and depression with past-week suicidal ideation and past-year suicide attempts. *N* = 6303. ACES, Adverse childhood experiences; PACES, Protective and compensatory childhood experiences. Values are standardized regression coefficients representing the direct associations between each variable. Suicidal ideation (past week) and suicide attempts (past year) were assessed at the 12-month follow-up and are dichotomous outcome variables (0 = No, 1 = Yes). Current symptoms of thwarted belongingness, perceived burdensomeness, and depression were assessed at baseline and are continuous mediator variables. ACES and PACES were assessed at baseline, and participants provided retrospective accounts of ACES and PACES that occurred on or before their 18^th^ birthday. ACES and PACES are continuous predictor variables. All paths were adjusted for age, race/ethnicity, employment, income, and education. * p ⩽ 0.05; ** p ⩽ 0.01; *** p ⩽ 0.001.

Results of our multivariate path analyses are displayed in Fig. 1 and estimates of direct associations of ACES and PACES with thwarted belongingness, perceived burdensomeness, and depression are presented in Table 2. ACES were positively associated with baseline thwarted belongingness (*β* = 0.15, *p* < 0.001), perceived burdensomeness (*β* = 0.19, *p* < 0.001), and depression (*β* = 0.22, *p* < 0.001). PACES were negatively associated with baseline thwarted belongingness (*β* = −0.30, *p* < 0.001), perceived burdensomeness (*β* = −0.18, *p* < 0.001), and depression (*β* = −0.18, *p* < 0.001).

**Table 2.** Results from multivariate path analysis estimating direct associations of ACES and PACES with thwarted belongingness, perceived burdensomeness, and depression

	Thwarted belongingness^a^			Perceived burdensomeness^a^			Depression^a^		
Variable	*β*^b^	95% CI^b^	*p* Value	*β*^b^	95% CI^b^	*p* Value	*β*^b^	95% CI^b^	*p* Value
Direct Effects									
ACES	0.15	(0.13 to 0.17)	<0.001	0.19	(0.16 to 0.21)	<0.001	0.23	(0.20 to 0.25)	<0.001
PACES	−0.30	(−0.32 to −0.27)	<0.001	−0.18	(−0.21 to −0.15)	<0.001	−0.19	(−0.21 to −0.16)	<0.001

ACES, Adverse childhood experiences; PACES, Protective and compensatory childhood experiences; CI, confidence interval. All paths were adjusted for age, sexual orientation, employment status, education, and income.

*N* = 6303.

a ACES, PACES, perceived burdensomeness, thwarted belongingness, and depression are continuous variables.

b Values are standardized beta coefficients and bootstrap confidence intervals with 10 000 replications.

Direct and indirect associations of ACES and PACES with suicidal ideation and suicide attempts are presented in Table 3. In terms of suicidal ideation, ACES were directly associated with higher odds of past-week suicidal ideation (AOR = 1.06, 95% CI 1.02–1.11) and indirectly associated with higher odds of past-week suicidal ideation via perceived burdensomeness and depression. Each increase in ACES was associated with 3% higher odds of past-week suicidal ideation at follow-up via higher perceived burdensomeness (AOR = 1.03, 95% CI 1.02–1.04) and 4% higher odds via higher depression (AOR = 1.04, 95% CI 1.03–1.06). The combined direct and indirect effects indicate that each increase in ACES was associated with 15% higher odds of past-week suicidal ideation at follow-up (AOR = 1.15, 95% CI 1.10–1.20). PACES were directly associated with lower odds of past-week suicidal ideation (AOR = 0.93, 95% CI 0.88–0.98) and indirectly associated with a lower odds of past-week suicidal ideation via perceived burdensomeness and depression. Each increase in PACES was associated with 4% lower odds of past-week suicidal ideation at follow-up via lower perceived burdensomeness (AOR = 0.96, 95% CI 0.95–0.97) and 4% via lower odds via lower depression (AOR = 0.96, 95% CI 0.95–0.97). The combined direct and indirect effects indicate that each increase in PACES was associated with 15% lower odds of past-week suicidal ideation at follow-up (AOR = 0.85, 95% CI 0.81–0.90).

**Table 3.** Results of multivariate path analysis estimating direct and indirect associations of ACES, PACES, thwarted belongingness, perceived burdensomeness, and depression with past-week suicidal ideation and past-year suicide attempt

	Suicidal ideation^a^^,b^ (past week)					Suicide attempt^a^ (past year)				
	*β*^c^	95% CI^c^	*p* Value	AOR	95% CI^c^	*β*^c^	95% CI^c^	*p* Value	AOR	95% CI^c^
*Direct effects*										
ACES	0.07	(0.02 to 0.12)	0.008	1.06	(1.02 to 1.11)	0.12	(0.03 to 0.20)	0.010	1.10	(1.02 to 1.19)
PACES	−0.07	(−0.12 to −0.02)	0.007	0.93	(0.88 to 0.98)	−0.02	(−0.11 to 0.07)	0.650	0.98	(0.89 to 1.08)
Thwarted belongingness	0.04	(−0.04 to 0.11)	0.335	1.01	(0.99 to 1.02)	0.06	(−0.07 to 0.20)	0.374	1.01	(0.99 to 1.03)
Perceived burdensomeness	0.21	(0.16 to 0.26)	<0.001	1.06	(1.04 to 1.08)	0.25	(0.17 to 0.33)	<0.001	1.07	(1.05 to 1.10)
Depression	0.22	(0.15 to 0.29)	<0.001	1.07	(1.05 to 1.10)	0.13	(0.01 to 0.25)	0.027	1.04	(1.01 to 1.08)
*Indirect effects*										
ACES via thwarted Belongingness	0.01	(−0.01 to 0.02)	0.337	1.01	(1.00 to 1.01)	0.01	(−0.01 to 0.03)	0.337	1.01	(0.99 to 1.03)
ACES via perceived burdensomeness	0.04	(0.03 to 0.05)	<0.001	1.03	(1.02 to 1.04)	0.05	(0.03 to 0.06)	<0.001	1.04	(1.03 to 1.06)
ACES via depression	0.05	(0.03 to 0.07)	<0.001	1.04	(1.03 to 1.06)	0.03	(0.00 to 0.06)	0.029	1.03	(1.00 to 1.05)
PACES via thwarted belongingness	−0.01	(−0.03 to 0.01)	0.336	0.99	(0.97 to 1.01)	−0.02	(−0.06 to 0.02)	0.375	0.98	(0.94 to 1.02)
PACES via perceived burdensomeness	−0.04	(−0.05 to −0.03)	<0.001	0.96	(0.95 to 0.97)	−0.04	(−0.06 to −0.03)	<0.001	0.96	(0.94 to 0.97)
PACES via depression	−0.04	(−0.06 to −0.03)	<0.001	0.96	(0.95 to 0.97)	−0.02	(−0.05 to 0.00)	0.029	0.97	(0.95 to 1.00)
*Combined indirect effects*										
ACES	0.10	(0.08 to 0.11)	<0.001	1.08	(1.07 to 1.10)	0.09	(0.06 to 0.11)	<0.001	1.08	(1.05 to 1.10)
PACES	−0.09	(−0.11 to −0.07)	<0.001	0.92	(0.90 to 0.93)	−0.09	(−0.12 to −0.05)	<0.001	0.92	(0.88 to 0.95)
*Total effects* (*Indirect plus direct*)										
ACES	0.16	(0.11 to 0.21)	<0.001	1.15	(1.10 to 1.20)	0.20	(0.12 to 0.28)	<0.001	1.19	(1.11 to 1.28)
PACES	−0.16	(−0.21 to −0.11)	<0.001	0.85	(0.81 to 0.90)	−0.11	(−0.19 to 0.11)	0.014	0.90	(0.82 to 0.98)

ACES, Adverse childhood experiences; PACES, Protective and compensatory childhood experiences; AOR, Adjusted odds ratio; CI, confidence interval. All paths were adjusted for age, sexual orientation, employment status, education, and income.

*N* = 6303.

a Suicidal ideation (past week) and suicide attempt (past year) are dichotomous variables (Yes/No).

b ACES, PACES, perceived burdensomeness, thwarted belongingness, and depression are continuous variables.

c Values are standardized beta coefficients and bootstrap confidence intervals with 10 000 replications.

For suicide attempts, ACES were directly associated with higher odds of a past-year suicide attempt (AOR = 1.10, 95% CI 1.02–1.19) and indirectly associated with higher odds of past-year suicide attempt via perceived burdensomeness. Each increase in ACES was associated with 4% higher odds of a past-year suicide attempt at follow-up via higher perceived burdensomeness (AOR = 1.04, 95% CI 1.03–1.06). The combined direct and indirect effects indicate that each increase in ACES was associated with 19% higher odds of a past-year suicide attempt (AOR = 1.19, 95% CI 1.11–1.28). The direct association of PACES with a past-year suicide attempt (AOR = 0.98, 95% CI 0.89–1.08) was not significant, but a significant indirect association was observed via perceived burdensomeness. Each increase in PACES was associated with 4% lower odds of a past-year suicide attempt via lower perceived burdensomeness (AOR = 0.96, 95% CI 0.94–0.97). The combined direct and indirect effects indicate that each increase in PACES was associated with 10% lower odds of a past-year suicide attempt (AOR = 0.90, 95% CI 0.82–0.98).

## Discussion

We examined the prospective associations of ACES, PACES, thwarted belongingness, and perceived burdensomeness with suicidality (e.g. suicidal ideation, suicide attempts) in a national longitudinal cohort of SMM. In this study, ACES were associated with higher thwarted belongingness and perceived burdensomeness, and PACES were associated with lower thwarted belongingness and perceived burdensomeness. Perceived burdensomeness was associated with a higher subsequent risk for suicidal ideation and suicide attempt. Significant indirect associations were observed for ACES and PACES with suicidal ideation and suicide attempts via perceived burdensomeness (but not thwarted belongingness). Our findings suggest ACES are associated with a higher risk of suicidality and PACES are associated with a lower risk of suicidality and that these associations are mediated, in part, by perceived burdensomeness.

Perceived burdensomeness is an important predictor of suicidal ideation and suicide attempts for SMM. Consistent with the Interpersonal-Psychological Theory of Suicide (Chu et al., 2017), we found that perceived burdensomeness was associated with a higher risk for past-week suicidal ideation and a past-year suicide attempt at 12-month follow-up. Our findings align with previous cross-sectional studies involving sexual minorities that demonstrated positive associations between perceived burdensomeness and suicidal ideation and suicide attempts (Baams et al., 2015; Hill & Pettit, 2012; Pate & Anestis, 2020; Ploderl et al., 2014; Woodward et al., 2014). Prior research has also shown that sexual minorities experience higher rates of perceived burdensomeness than heterosexual populations (Hill & Pettit, 2012; Pate & Anestis, 2020), suggesting perceived burdensomeness may contribute to observed disparities in suicidal ideation and suicide attempts among SMM.

In most studies, when examined alongside perceived burdensomeness, thwarted belongingness has not been associated with suicidal ideation or suicide attempts (Chu et al., 2017). Prior cross-sectional samples of U.S. sexual minority adults (Woodward et al., 2014), college students (Hill & Pettit, 2012), and youth have produced similar results. In a study of youth, Baams et al. (2015) failed to find an association among young SMM. Still, they did find a small but significant positive association among the young sexual minority women in their sample. Yet one study of Bavarian sexual minority adults reported significant positive associations between thwarted belongingness and suicidal ideation and suicide attempts (Ploderl et al., 2014). Although future research should not rule out the possibility of cultural differences in the meaning and role of thwarted belongingness in suicidality, taken together, the body of research suggests that for U.S. SMM, thwarted belongingness is not a significant predictor of suicidality.

The negative impact of ACES on mental health outcomes is well-documented (Sahle et al., 2021); SMM who reported high ACES reported higher thwarted belongingness and perceived burdensomeness and were at higher risk for suicidal ideation and a suicide attempt. In contrast, our findings offer novel evidence that PACES may protect against suicidality into adulthood. PACES were associated with lower thwarted belongingness, perceived burdensomeness, and lower risk for suicidal ideation and suicide attempts. Further, mediation analyses suggest that perceived burdensomeness mediated the effects of ACES and PACES on suicidal ideation and suicide attempts. These findings on ACES are concerning given the disproportionately high prevalence of ACES in sexual minority populations (Andersen & Blosnich, 2013). Yet, results for PACES offer hope that individuals who also experienced some PACES would be at lower risk for suicidality in adulthood.

Taken together, our results suggest that the ACES and PACES have an important influence on thwarted belongingness and perceived burdensomeness in adulthood and that suicide risk among SMM may be lowered by assessing and treating perceived burdensomeness in this population. Given the high prevalence of ACES among SMM, providers should consider evaluating for perceived burdensomeness among all SMM and using cognitive-behavioral interventions adapted explicitly for this population to reduce these perceptions and associated suicide risk. For example, a cognitive-behavioral intervention was adapted for young SMM that focused on restructuring maladaptive cognitions rooted in sexual minority stigma and building supportive and affirming relationships to improve depression, substance use, and HIV transmission risk outcomes (Pachankis, Hatzenbuehler, Rendina, Safren, & Parsons, 2015). This intervention may be helpful in addressing cognitions surrounding burdensomeness and improving the quality of relationships among SMM at risk for suicide.

Future research should compare the effects of perceived burdensomeness on suicide risk to other psychosocial risk factors. Additionally, future studies should routinely incorporate ACES when assessing risk factors among sexual minority adults and youth. PACES should be included to understand protective factors and analyze their mitigating impact of ACES on suicide risk and behaviors among both sexual minority youth and adults. Though it is unclear just how modifiable ACES and PACES are in particularly high-risk childhood environments at the individual level (Lorenc, Lester, Sutcliffe, Stansfield, & Thomas, 2020), there may be an opportunity for future research in this area to guide community and policy-level advocacy work that seeks to provide resources and community aid to children most at-risk for experiencing ACES. Programs aimed at reducing ACES and increasing PACES for sexual minority youth would likely have positive effects on the health of suicide and other health outcomes in future generations of SMM.

### Limitations

The results of our study should be considered in the context of some limitations. First, we relied on retrospective self-reports of ACES and PACES, which may have been subject to recall bias. Further, power may have been compromised due to the relatively low occurrence of suicidal ideation and suicide attempts, despite our large sample size. Conversely, large sample sizes and multiple comparisons increase the risk of Type I error, suggesting that small effects should be interpreted with caution. Although the sample was diverse, results may not generalize beyond SMM who met the study's eligibility criteria. Expressly, study enrollment was limited to SMM who used mobile apps to find sexual partners and reported engagement in HIV risk behavior in the past six months, potentially resulting in selection bias. Although research suggests that a majority of SMM (67%) used mobile apps to meet other men in the past year (Hecht, Zlotorzynska, Sanchez, & Wohlfeiler, 2022), use of these apps is associated with a lower sense of community and higher loneliness (Zervoulis, Smith, Reed, & Dinos, 2020). Suicidal ideation has also been associated with HIV risk behavior in prior studies of SMM (Halkitis et al., 2015). Future research should attempt to replicate the present study's findings among SMM who did not meet eligibility criteria.

A limited number of studies have observed differences in ACES and availability of PACES at the intersection of different minority identities separately (English et al., 2022), such as among Black and Latinx communities and transgender and gender-diverse populations (Brown, 2008; Maguire-Jack, Lanier, & Lombardi, 2020; Schnarrs et al., 2019). Future research should also identify differences in ACES and PACES at the intersection of race/ethnicity, gender identity, and sexual identity as unique predictors of suicidality among these populations. Finally, the Interpersonal-Psychological Theory of Suicide focuses on the crucial role of thwarted belongingness and perceived burdensomeness as preconditions of suicidality. Yet, the theory includes mechanisms that moderate the link between these preconditions and suicidality (e.g. acquired capacity; Anestis, Moberg, & Arnau, 2014) and has been extended to include how thwarted belongingness and perceived burdensomeness may mediate the association between hopelessness and suicide ideation (Kleiman, Law, & Anestis, 2014). Future research should aim to extend the Interpersonal-Psychological Theory of Suicide for understanding suicidality among SMM.

## Conclusions

This study establishes that ACES are associated with higher suicide risk via higher perceived burdensomeness and that PACES may provide some protection against suicide by reducing perceived burdensomeness. Our findings suggest that for SMM, screening for perceived burdensomeness is recommended to identify men at risk for suicide. Providers should consider using cognitive behavioral interventions adapted for SMM to reduce perceived burdensomeness and associated suicide risk. Additional research is needed to identify ways to minimize ACES and increase PACES for sexual minority youth, potentially benefitting the health of future generations of SMM.

## Supplementary material

For supplementary material accompanying this paper visit https://doi.org/10.1017/S0033291722002823. click here to view supplementary material
