# Supplementary material for: Associations of adverse and protective childhood experiences with thwarted belongingness, perceived burdensomeness, and suicide risk among sexual minority men
Source: Psychol Med. 2022 Sep 19;53(12):5615–24. doi: 10.1017/S0033291722002823 (PMC10024646; doi:10.1017/S0033291722002823)
Supplement: Supplementary file 1 [file S0033291722002823sup001.docx]

| Table S1. Descriptive statistics and bivariate associations for predictor, mediator, and outcome variables | | | | | | | |
| --- | --- | --- | --- | --- | --- | --- | --- |
|  | 1 | 2 | 3 | 4 | 5 | Suicidal Ideation  (past week) ^b^ | Suicide Attempt  (past year) ^b^ |
|  | *r* ^c^ | | | | | *t ^d^* | |
| 1. ACES ^a^ | ─ |  |  |  |  | 12.73 | 24.32 |
| 2. PACES ^a^ | -0.29 | ─ |  |  |  | -10.51 | -11.22 |
| 3. Thwarted Belongingness ^a^ | 0.24 | -0.34 | ─ |  |  | 35.51 | 24.36 |
| 4. Perceived Burdensomeness ^a^ | 0.26 | -0.24 | 0.60 | ─ |  | 23.09 | 16.05 |
| 5. Depression ^a^ | 0.29 | -0.25 | 0.63 | 0.55 | ─ | 29.43 | 20.48 |
| Mean | 2.44 | 7.48 | 26.88 | 10.73 | 11.18 |  |  |
| Median | 2.00 | 8.00 | 26.00 | 7.00 | 11.00 |  |  |
| SD | 2.39 | 2.03 | 12.40 | 7.18 | 6.44 |  |  |
| IQR | 0.0-4.0 | 6.0-9.0 | 17.0-36.0 | 6.0-13.0 | 5.0-16.0 |  |  |
| N = 6,303. Abbreviations: ACES, Adverse childhood experiences; PACES, Protective and compensatory childhood experiences. ^a^ ACES, PACES, perceived burdensomeness, thwarted belongingness, and depression are continuous variables. ^b^ Suicidal ideation (past week) and suicide attempt (past year) are dichotomous variables (Yes/No). ^c^ Values are Pearson correlation coefficients. ^d^ Values are independent sample t-tests and each test had 6,301 degrees of freedom. *P* < .001 for all associations. | | | | | | | |
